# Enhanced histamine production through the induction of histidine decarboxylase expression by phorbol ester in Jurkat cells

**DOI:** 10.3892/mmr.2012.1049

**Published:** 2012-08-27

**Authors:** YUSUKE NAGASHIMA, KOICHIRO KAKO, JUN-DAL KIM, AKIYOSHI FUKAMIZU

**Affiliations:** 1 Graduate School of Life and Environmental Sciences, University of Tsukuba, Tsukuba, Ibaraki 305-8577, Japan; 2 Faculty of Life and Environmental Sciences, University of Tsukuba, Tsukuba, Ibaraki 305-8577, Japan; 3 Life Science Center of Tsukuba Advanced Research Alliance (TARA), University of Tsukuba, Tsukuba, Ibaraki 305-8577, Japan

**Keywords:** histamine, histidine decarboxylase, Jurkat cells, phorbol 12-myristate 13-acetate, matrix-assisted laser desorption/ionization quadrupole ion trap time-of-flight tandem mass spectrometry, ultra-high performance liquid chromatography

## Abstract

Histamine (HA), a mediator of inflammation, type I allergic responses and neurotransmission, is synthesized from L-histidine, the reaction of which is catalyzed by histidine decarboxylase (HDC). HDC has been reported to be induced by various stimuli, not only in mast cells and basophils, but also in T lymphocytes and macrophages. Although its mRNA has been shown to be increased in Jurkat cells when treated with phorbol 12-myristate 13-acetate (TPA), little is known concerning the induced production of HA by HDC. The present study quantified the trace amounts of intracellular HA using ultra-high liquid chromatography in combination with the 6-aminoquinoline carbamate-derivatization technique. To test whether the cellular level of HA is elevated by the induction of HDC in Jurkat cells treated with TPA, the peak corresponding to authentic HA in the cell lysate was fractioned and its molecular weight determined by matrix-assisted laser desorption/ionization quadrupole ion trap time-of-flight mass spectrometry. The results of this study show that the HA level is increased by the induction of HDC expression by TPA in Jurkat cells. Therefore, this method is useful in elucidating the physiological significance of HA production.

## Introduction

Histamine (HA) is involved in a variety of physiological processes, including inflammation, allergic responses, neurotransmission and gastric acid secretion (1,2). HA is synthesized by a pyridoxal phosphate-dependent enzyme, histidine decarboxylase (HDC, EC 4.1.1.22), with L-histidine as a substrate. The genetic expression of HDC has been widely detected throughout the human body (3,4). HDC is reportedly induced by various stimuli, not only in mast cells and basophils, but also in T lymphocytes and macrophages (5,6). In particular, this non-mast cell- and/or non-basophil-derived HA has been shown to be involved in the regulation of angiogenesis, inflammatory granulation and cell proliferation (6,7). It has been demonstrated that cultured T lymphocytes overexpressed the HDC gene in response to concanavalin A or various cytokines and secreted HA into a culture medium (5,8).

In Jurkat cells, a model human cell line for T cells used in numerous studies, HDC gene transcription was markedly enhanced by treatments with phorbol 12-myristate 13-acetate (TPA) or phytohemagglutinin (PHA) (9). However, no studies assessing HA production in TPA-stimulated Jurkat cells are currently available. In the current study, a rapid and high-sensitivity derivatization technique was introduced, used with 6-aminoquinoline carbamic acid (AQC) as a fluorescent reagent (10,11), for detecting the trace amounts of intracellular HA. The cellular level of HA in Jurkat cells treated with TPA was then determined by developing an accurate and reliable method quantified by ultra-high performance liquid chromatography (UPLC) in combination with matrix-assisted laser desorption/ionization quadrupole ion trap time-of-flight tandem mass spectrometry (MALDI-QIT-TOF/MS) for the molecular identification of HA.

## Materials and methods

### Chemicals and reagents

HA, 3-methylhistamine (3-methylHA) dihydrochloride, (R)(−)-α-methylHA dihydrochloride and TPA were purchased from Sigma-Aldrich (St. Louis, MO, USA). HPLC-grade methanol and acetonitrile were obtained from Wako (Tokyo, Japan). An AccQ-Tag Ultra-Fluor™ derivatization kit (borate buffer and reagent) was purchased from Waters (Milford, MA, USA). Bradykinin fragment 1–7, the calibration standard for MALDI-QIT-TOF/MS, was purchased from Sigma-Aldrich. 2,5-Dihydroxybenzoic acid (DHBA) matrix of MALDI-MS grade was purchased from Shimadzu GLC (Kyoto, Japan).

### Cell culture

Jurkat cells, a human leukemic T-cell line, were maintained in RPMI 1640 complete medium (Wako) with 10% heat-inactivated fetal bovine serum (FBS, Invitrogen, Carlsbad, CA, USA), 2% (w/v) L-glutamine, 100 U/ml penicillin and 100 mg/ml streptomycin (Invitrogen). HeLa S3 cells, a human epithelial carcinoma, were maintained in RPMI 1640 supplemented with 5% FBS. The two cell lines were cultured in a humidified atmosphere containing 5% CO_2_ at 37°C.

### RNA preparation and RT-PCR

Total RNA was extracted from cultured Jurkat cells (5×10^6^ cells/dish) and HeLa S3 cells (3×10^6^ cells/dish) treated with TPA (10 ng/ml) or dimethyl sulfoxide (DMSO) for 16 h. The synthesis of cDNA and RT-qPCR was performed as previously described (12). The primers used for amplification were *hdc* forward, 5′-CAAGCACATGTCAGAGATGG-3′; *hdc* reverse, 5′-TGAACAGGAAGGAGGACAG-3′; glyceraldehyde 3-phosphate dehydrogenase (*GAPDH*) forward, 5′-GTC TTCACCACCATGGAGAAGGC-3′; *GAPDH* reverse, 5′-GCAGTGATGGCATGGACTGTGGT-3′. The number of PCR cycles of amplification for each primer set was determined by carrying out preliminary experiments to avoid the saturation of PCR products. The sizes of the PCR products of *hdc* and *GAPDH* were 589 and 248 bp, respectively.

### Sample preparation and derivatization for chromatographic analysis

Jurkat cells were plated at 1.25×10^7^ cells/well and harvested following stimulation with DMSO or TPA (10 ng/ml) for 16 h. Cells were homogenized in 0.6-ml Milli-Q™ water using an ultrasonic cell disruptor on ice. Cell debris and insoluble matter were removed by centrifugation at 14,000 rpm for 10 min at 4°C. The protein concentrations of the lysates were determined by Bio-Rad Protein Assay reagent (Bio-Rad, Hercules, CA, USA) with aliquots of lysate. HA extraction was performed as previously described by Shore *et al* (13), with minor modifications. After adding 2 nmol of α-methylHA into each sample, samples were acidified with 60% perchloric acid and vortexed. The acid extract was maintained on ice for 10 min to precipitate proteins and was then centrifuged at 14,000 rpm for 20 min to remove the denatured proteins.

Supernatant (in 500-μl aliquots) was transferred to a 2-ml tube containing 0.5 ml of 5 N NaOH, 200 mg of solid NaCl and 1 ml of n-butanol. The tube was agitated for 5 min by shaker to extract the HA into the butanol. After centrifugation at 14,000 rpm for 10 min, the organic phase was transferred to the fresh tube and agitated for ~1 min with 500 μl of salt-saturated 0.1 N NaOH. The tube was then centrifuged at 14,000 rpm for 10 min and a 1-ml aliquot of organic phase was transferred to a 2-ml tube containing 375 μl of 0.1 N HCl and 700 μl of n-heptane. Subsequent to 5-min agitation, the tube was centrifuged at 14,000 rpm for 10 min and the HA in the aqueous phase was collected and dried with a rotary concentrator. HA derivatization with AccQ-Tag reagents was conducted as previously described (11). Briefly, the dried cell extract was dissolved with 10 μl of Milli-Q water combined with 10 μl of AccQ-Tag Ultra borate buffer. AccQ-Tag reagent (20 μl) previously dissolved in 1.0 ml of AccQ-Tag Ultra reagent diluent was added. After a 1-min incubation at room temperature, the reaction mixture was concentrated with a centrifugal evaporator.

### Chromatographic conditions

UPLC was performed on an Acquity™ UPLC system (Waters), equipped with an FLR fluorescent detection system. Fluorescent derivatives were separated on an AccQ-Tag™ amino acid analysis C18 (Waters; 1.7 μm, 2.1×100 mm) column with a VanGuard™ cartridge (Waters) at 40°C with an acetonitrile gradient in ammonium acetate buffer (pH 6.0) at a flow rate of 0.25 ml/min. Mobile phase solutions A and B were 4 and 60% acetonitrile in 0.1 M ammonium acetate, pH 6.0, respectively. The gradient conditions were: 0% B for 25 min, 0–3.5% B for 20 min, 3.5–6% B for 0.5 min, 6% B for 10 min, 6–7% B for 0.5 min, 7% B for 10 min, 7–8% B for 0.5 min and 8% B for 10 min. Following gradient elution, the column was rinsed with 100% B for 5 min, then returned to the initial conditions (0% B) in 0.5 min to allow the column to re-equilibrate for 10 min. The injection volume was 7.5 μl. Target peaks were fractioned and subsequently samples were dried completely using an evaporator.

### MALDI-QIT-TOF/MS

DHBA matrix (5 mg) was dissolved in 0.5 ml of 16% acetonitrile and 0.06% trifluoroacetic acid. Matrix solution (0.5 μl) and 0.5 μl of sample dissolved in Milli-Q water were deposited on a MALDI plate and left to dry at room temperature to prepare sample spots. External calibrations were achieved using the standard reagents of DHBA matrix (monoisotopic mass of [(M+H)^+^ = 155.03] and bradykinin fragment 1–7 (monoisotopic mass of [(M+H)^+^ = 757.4]). MS experiments with MALDI-QIT-TOF/MS^n^ (n=1, 2) were performed using argon as the collision gas on an AXIMA Resonance mass spectrometer (Shimadzu-Biotech, Kyoto, Japan) equipped with a nitrogen laser (337 nm), with the collision-induced dissociation (CID) control value set at 230.

### Standard curves

Calibration standards for HA (0, 0.25, 0.5, 1.0, 2.0 and 4.0 nmol) were prepared from the stock solution (25 μg/ml) diluted in Milli-Q water and stored at −20°C. For the preparation of the calibration curves, standards for HA and internal standards (2 nmol of α-methylHA) were added to cell lysates and subjected to extraction and chromatography as described above. Calibration curves were calculated by plotting the ratio of the peak area of analytes to the area of the internal standard (α-methylHA) vs. analyte quantity.

## Results

### Induction of HDC mRNA in Jurkat cells

**Figure 1:**
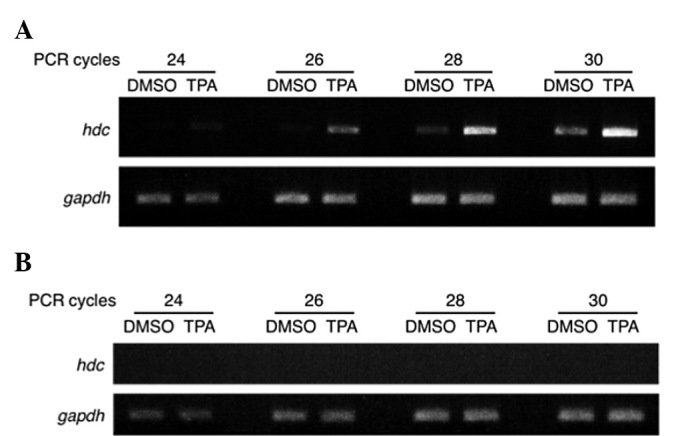
Effects of phorbol ester on HDC mRNA induction in cells. (A) Cultured Jurkat or (B) HeLa S3 cells were treated with TPA or DMSO for 16 h and levels of specific mRNAs for *hdc* were determined by quantitative RT-PCR. PCR cycle numbers are shown over each lane. *GAPDH* was used as an internal control. HDC, histidine decarboxylase; TPA, phorbol 12-myristate 13-acetate; DMSO, dimethyl sulfoxide.

To confirm a previous result (9), total RNA was extracted from Jurkat and HeLa S3 cells to be used as a negative control and the level of *hdc* mRNA was examined by an RT-PCR experiment using gene-specific primers for human *hdc*. As shown in Fig. 1A, the level of *hdc* mRNA was significantly increased when stimulated with TPA in Jurkat cells. Conversely, it was not detected in HeLa S3 cells with or without TPA (Fig. 1B).

### Development of methods

**Figure 2:**
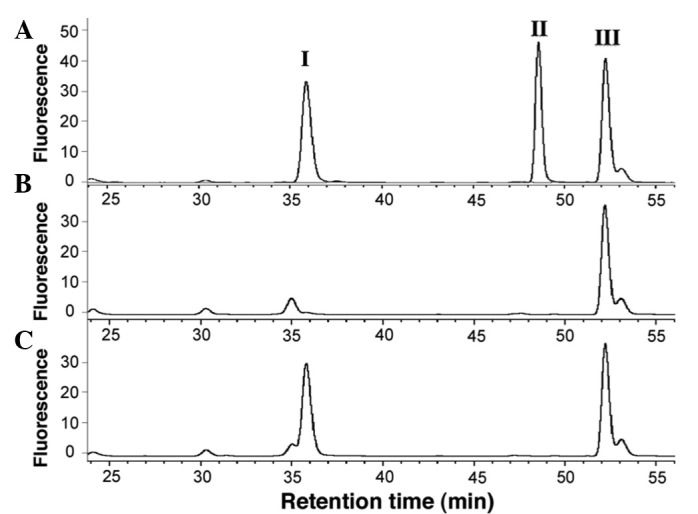
Chromatographic characterization of AQC-HA derivatives. (A) Representative UPLC chromatogram of a combined standard containing 200 pmol of HA (I) and 3-methylHA (II) and 2 nmol of (R)(−)-α-methylHA (III). Typical chromatogram of cell lysates obtained from Jurkat cells treated with (B) DMSO or (C) TPA. Chromatographic conditions are described in Materials and methods. AQC, amino quinoline carbamic acid; HA, histamine; UPLC, ultra-high performance liquid chromatography; DMSO, dimethyl sulfoxide; TPA, phorbol 12-myristate 13-acetate.

HA, 3-methylHA and α-methylHA were fluorescently derivatized with AQC as described in Materials and methods. Typical UPLC (equipped with ODS column) chromatograms were obtained following the injection of standard samples containing AQC-labeled HA-derivatives (Fig. 2A). Retention times of AQC-HA (peak I), AQC-3-methylHA (peak II) and AQC-α-methylHA (peak III) were 35.9, 48.6 and 52.3 min, respectively. Representative chromatograms of samples from non- and TPA-stimulated Jurkat cells are shown in Fig. 2B and C, respectively.

### MS and MS/MS

**Figure 3:**
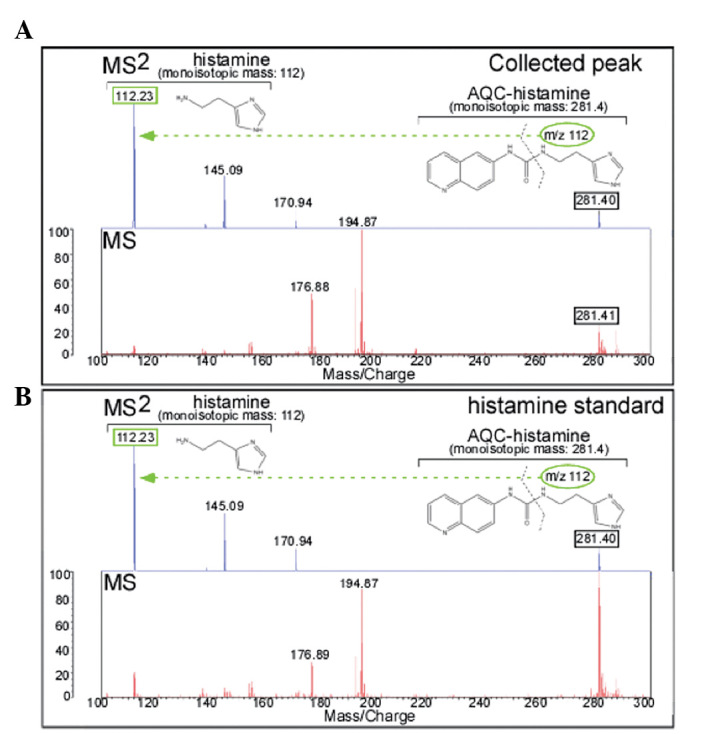
Molecular identification of the AQC-HA with MALDI-QIT-TOF/MS. (A) Typical MS ≥ MS/MS spectra of the collected peak eluted in an identical position to the AQC-standard HA in the lysate of Jurkat cells. (B) Representative MS ≥ MS/MS spectra of AQC-derivatized standard HA. The number of theoretical molecular masses (m/z) in the structural formula are shown with circles and those of experimental values are indicated in the boxes. The transitions of m/z are depicted with the green dashed arrows. AQC, amino quinoline carbamic acid; HA, histamine; MALDI-QIT-TOF/MS, matrix-assisted laser desorption/ionization quadrupole ion trap time-of-flight tandem mass spectrometry.

To assess the identity of the peak derived from Jurkat cells, the peak was fractioned with the retention time corresponding to that of the AQC-derivatized standard HA and mass analysis with MALDI-QIT-TOF/MS was carried out. Representative MS and MS/MS spectra of the putative AQC-HA [(M+H)^+^ = 281.4] derived from Jurkat cells were observed (Fig. 3A). Following CID, the parental ion produced the fragment ion 170.94 (the eliminated AQC-group). Specific daughter transition for AQC-HA≥HA was revealed to be of 112.23 molecular mass (m/z). Identical fragmentation patterns were observed in the MS and MS/MS spectra of AQC-standard HA (Fig. 3B), indicating that the peak in the lysate of TPA-treated Jurkat cells (Fig. 2C) was AQC-HA.

### Calibration and quantitative analyses

**Figure 4:**
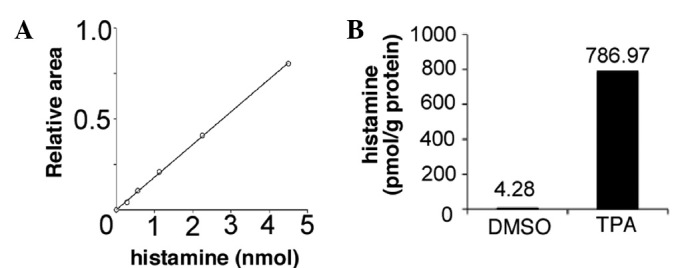
Quantitative analysis of cellular HA. (A) Standard curves for peak area ratio vs. weight for HA. Conditions were as in Fig. 2. (B) Cellular contents of HA in the cultured Jurkat cells treated with DMSO or TPA. Treatment conditions of cells are described in Materials and methods. HA, histamine; DMSO, dimethyl sulfoxide; TPA, phorbol 12-myristate 13-acetate.

A calibration curve was calculated using the peak area vs. concentrations of the standard analyte. The linearity was verified over the assay range (0–4.0 nmol on-column). The typical linear regression equation from the standard curve for HA was *y*=5.013×10^5^*x* where *y* was the peak area ratio of analyte to internal standard and *x* the analyte amount (nmol). The correlation coefficient for the standard in cell lysates was 0.999 (Fig. 4A). Under this condition, the intracellular HA was measured and the increase in its content from 4.28 (untreated) to 786.97 pmol/mg protein (treated with TPA) was identified (Fig. 4B) through the induction of the HDC gene expression (Fig. 1A).

## Discussion

The transcriptional activation of HDC has been proven to be controlled by the gastrin-responsive element located in the 5′-region of its genome (14). Moreover, treatment with TPA and/or PHA enhances this promoter activity in Jurkat cells (9). However, little is known about the induced production of HA in cultured cells. The present study demonstrates that the production of HA was upregulated in association with HDC induction stimulated by TPA in Jurkat cells.

Quantitative analyses of HA by HPLC have been previously reported using fluorescence derivatization with dansyl chloride (15) or *o*-phthalaldehyde (13). However, these conventional methods are too time-consuming to complete the reaction (15) or require the acidification of the reaction mixture to stabilize the fluorophore (13). The current study adopted a sensitive and accurate method for quantifying HA by UPLC combined with an AQC-labeling technique (16–18), which is a more rapid and stable method than those used previously; the derivatization is completed in under 1 min and AQC-derivatives are immediately applied to the UPLC system. By using the elution program described in Materials and methods, standard AQC-HA (peak I) was observed as the single sharp peak and AQC-α-methylHA (peak III) was also detected to allow accurate integration for quantification as an internal standard (Fig. 2A).

A significantly increased peak was identified at an identical position to the AQC-standard HA in the lysates of the Jurkat cells treated under conditions described in Fig. 1A (Fig. 2C), with this peak being collected for MS analysis. Since the MS ≥ MS/MS sequential analysis with MALDI-QIT-TOF/MS revealed that the CID patterns of the UPLC-fractioned peak (Fig. 3A) were identical to those of the AQC-derivatized standard HA (Fig. 3B), we concluded that the substance increased by the TPA-stimulation in Jurkat cells was HA. Since the linear regression analysis demonstrated significant linearity of the calibration curve from 0 to 4.0 nmol (Fig. 4A), the intracellular HA was measured with this calibration curve and revealed that the amount of HA had increased to approximately 180-fold by TPA, compared with the non-stimulated cells (Fig. 4B).

HA is metabolized in two ways: one is the oxidative deamination by diamine oxidase (DAO, EC 1.4.3.22) and the other is imidazole ring-methylation by histamine-*N*-methyltransferase (HNMT, EC 2.1.1.8) that converts HA to 3-methylHA only in the cytoplasm (19–21). No peaks were detected in Fig. 2 corresponding to the standard 3-methylHA derivatized with AQC in TPA-stimulated cells and/or control cells, although the HNMT gene was expressed in the Jurkat cells treated with or without TPA (data not shown). The reason for 3-methylHA not being detected in the HDC-induced cells may be as follows: i) low contents of 3-methylHA below measurable limits; ii) ineffective translational rates of HNMT; iii) inactivation or degradation of HNMT; or iv) rapid secretion of 3-methylHA into culture media.

Results of a previous study have demonstrated that the overproduced HA in T lymphocytes stimulated by concanavalin-A was secreted to the extracellular space (8). However, the mechanisms of secretion and/or metabolism remain to be determined. Therefore, additional investigation of the culture medium is required for the estimation of the total cellular production of HA in T lymphocytes.

In conclusion, the method described in this study is useful for elucidating the physiological significance of HA production, not only in T lymphocytes, but also in other cell types.
